# The Co-Existence of Hypovitaminosis D and Diabetes Mellitus Triples the Incidence of Severe Coronary Artery Disease in Women

**DOI:** 10.3390/jcm13226792

**Published:** 2024-11-12

**Authors:** Aneta Aleksova, Milijana Janjusevic, Beatrice Pani, Cristina Hiche, Andrea Chicco, Agnese Derin, Lorenzo Zandonà, Elisabetta Stenner, Daria Beltrame, Marco Gabrielli, Stefano Lovadina, Flávia Campos Corgosinho, Stefano D’Errico, Maria Marketou, Donna R. Zwas, Gianfranco Sinagra, Alessandra Lucia Fluca

**Affiliations:** 1 Cardiothoracovascular Department, Azienda Sanitaria Universitaria Giuliano Isontina, 34100 Trieste, Italy; mjanjusevic@units.it (M.J.); cristina.hiche@asugi.sanita.fvg.it (C.H.); agnese.derin@asugi.sanita.fvg.it (A.D.); daria.beltrame@asugi.sanita.fvg.it (D.B.); gianfranco.sinagra@asugi.sanita.fvg.it (G.S.); alessandralucia.fluca@units.it (A.L.F.); 2 Department of Medical Surgical and Health Sciences, University of Trieste, 34125 Trieste, Italy; beatrice.pani@phd.units.it (B.P.); marco.gabrielli@studenti.units.it (M.G.); sderrico@units.it (S.D.); 3 SC Laboratorio Unico, Ospedale Maggiore, Azienda Sanitaria Universitaria Giuliano Isontina, 34125 Trieste, Italy; andrea.chicco@asugi.sanita.fvg.it (A.C.); lorenzo.zandona@asugi.sanita.fvg.it (L.Z.); 4 Department of Diagnostics, Azienda USL Toscana Nordovest, 56121 Livorno, Italy; elisabetta.stenner@uslnordovest.toscana.it; 5 Department of General and Thoracic Surgery, Cattinara University Hospital, 34149 Trieste, Italy; stefano.lovadina@asugi.sanita.fvg.it; 6 Faculty of Nutrition, Federal University of Goias, Goiânia 74605-050, Brazil; flaviacorgosinho@ufg.br; 7 Cardiology Department Crete, School of Medicine, Heraklion University General Hospital, University of Crete, 70013 Iraklio, Greece; maryemarke@yahoo.gr; 8 Linda Joy Pollin Cardiovascular Wellness Center for Women, Heart Institute, Hadassah University Medical Center, Jerusalem 91120, Israel; donnaz1818@gmail.com

**Keywords:** vitamin D, hypovitaminosis D, gender difference, acute myocardial infarction, coronary artery disease

## Abstract

**Background and Aims:** Hypovitaminosis D is involved in the development and progression of atherosclerosis, and it is more prevalent in women. The differential impact of hypovitaminosis D on the severity of coronary artery disease (CAD) between genders remains poorly understood. This study aims to address this literature gap. **Methods:** A total of 1484 consecutive patients with acute myocardial infarction (AMI) were enrolled in the study. Hypovitaminosis D was defined as vitamin D ≤ 20 ng/mL. CAD was defined as the presence of at least one coronary vessel stenosis > 50%, while severe CAD was defined as left main disease and/or three-vessel disease > 50%. **Results:** The mean age of the cohort was 66.3 (11.5) years, with a predominance of the male gender (71.8%). Vitamin D values were significantly lower in women than in men (15.7 [8.4–25.4] ng/mL vs. 17.9 [11–24.3] ng/mL, *p* = 0.01). A higher prevalence of severe CAD was observed in female patients with hypovitaminosis D compared to those without (33% vs. 19%, *p* < 0.01). This finding was not observed in men. Among women, hypovitaminosis D significantly increased the risk of severe CAD (OR: 1.85, *p* = 0.01), together with diabetes mellitus (DM) and older age, adjusted for GFR < 60 mL/min/1.73 m^2^, cholesterol and body mass index. Furthermore, women with both hypovitaminosis D and DM had more than three times the risk of severe CAD compared with women who lacked both (OR: 3.56, *p* = 0.02). **Conclusions:** In women, hypovitaminosis D increases the risk of severe CAD, and the co-existence of hypovitaminosis D and DM triples the incidence of severe CAD.

## 1. Introduction

Coronary artery disease (CAD) is the leading cause of morbidity and mortality worldwide despite advances in the diagnosis and treatment of this condition. More precisely, the most recent statistical data indicate that CAD represents the third most significant cause of mortality, with a considerable economic burden [1]. As life expectancy rises, the prevalence of CAD is expected to increase. Consequently, the scientific community is currently prioritising the development of preventative measures with the objective of avoiding the onset of the disease. A key strategy is to enhance public consciousness about the deleterious consequences of modifiable risk factors, particularly smoking, poor dietary habits, and a sedentary lifestyle. Therefore, adopting a healthy lifestyle can prevent or delay the onset of CAD, even in individuals with a family history of cardiovascular disease. Furthermore, it can slow the progression of CAD in patients who have already been diagnosed with the disease.

In particular, hypovitaminosis D (vitamin D ≤ 20 ng/mL) is recognised as a modifiable risk factor, which is associated with a number of serious health conditions, including CAD and its extent, chronic kidney disease (CKD) and metabolic disorders, such as diabetes mellitus (DM), insulin resistance and obesity [2,3,4]. Moreover, in our previous paper, we demonstrated that hypovitaminosis D has comparable effects to those of DM in terms of mortality risk among patients with acute myocardial infarction (AMI) [5]. The rationale for this is that vitamin D has cardioprotective properties and regulates a multitude of processes, including oxidative stress, inflammation, endothelial function and platelet aggregation [2,4]. Consequently, the deficiency of this hormone has been identified as a factor associated with the development and progression of atherosclerosis, as well as vascular calcifications and stiffness [2,4]. For these reasons, it is important to maintain optimal levels of vitamin D. Also, it has been observed that the production of vitamin D in the skin declines by approximately 13% per decade in both genders [6], but the prevalence of hypovitaminosis D is higher among women compared to men [7,8,9]. There is evidence that hypoestrogenism during the postmenopausal period is associated with the impaired expression of vitamin D receptors and reduced intestinal absorption of vitamin D from food sources [10], which may explain this gender discrepancy [7,10]. Unfortunately, there is a paucity of research examining the potential discrepancy between genders in the correlation between hypovitaminosis D and CAD [2,3].

Therefore, the objective of this study is to examine the potential role of hypovitaminosis D as a risk factor for the prevalence of CAD and its severity, focusing on gender differences in a real-world cohort of patients hospitalised for AMI.

This study aims to contribute new data and insights to the limited existing literature on gender discrepancies in this area and gain a deeper understanding of the nature of the relationship itself.

## 2. Materials and Methods

### 2.1. Study Population and Data Sources

A total of 1484 consecutive patients diagnosed with AMI, comprising both ST Elevation Myocardial Infarction (STEMI) and Non-ST Elevation Myocardial Infarction (NSTEMI), who were hospitalised at the Department of Cardiology of the University Hospital of Trieste between 2009 and 2022 and satisfied the inclusion criteria, were enrolled in this study. The inclusion criteria required that the symptoms of AMI had started within 24 h and that the patients were of legal age (>18 years old). The project was approved by our local ethics committee (No. 67/2015, update CEUR-2021-Em-220 dd. 22/06/2021 PROT 0025024/P/GEN/ARCS) and was performed in accordance with the Declaration of Helsinki.

For the purposes of this prospective study, the baseline characteristics of the patients at the time of hospital admission, pharmacological treatment, procedures, hospital course and outcomes, patient medical history and family history were collected using the electronic health record software Cardionet (INSIEL 2.5, Trieste, Italy) and G2 Clinical (INSIEL, Trieste, Italy). We were able to collect all data of interest, and no patients were excluded. Furthermore, none of the patients included in the study had been prescribed or had initiated vitamin D supplementation prior to the ischemic event.

### 2.2. Definitions

AMI was diagnosed according to the European Society of Cardiology (ESC) guidelines [11]. CAD was defined as the presence of at least one coronary vessel stenosis greater than 50%, whereas severe CAD was defined as three-vessel and/or left main coronary artery disease with >50% stenosis [2]. CKD was defined as a glomerular filtration rate (GFR) less than 60 mL/min/1.73 m^2^ [12]. Hypovitaminosis D and severe hypovitaminosis D were defined as vitamin D ≤ 20 ng/mL and ≤10 ng/mL, respectively [4]. The Killip and GRACE scores were defined in accordance with the previously established definitions [13,14].

### 2.3. Sample Collection and Analysis

Blood samples were taken at hospital admission and collected in K2 ethylenediaminetetraacetic acid from all patients. Prior to centrifugation, which was performed within eight hours, the samples were stored at 4 °C. Subsequently, the samples were centrifuged at 2500× *g* for 15 min at +4 °C to isolate the plasma, which was stored at −80 °C until further analysis.

To assess the plasma vitamin D status of patients with AMI, a chemiluminescence assay using the Liaison^®^ instrument (DiaSorin Inc., Saluggia, Italy) was applied to measure the 25-hydroxyvitamin D [25(OH)D] levels. 25(OH)D is an inactive form of vitamin D that is routinely used to assess the status of this hormone due to its long half-life of 2–3 weeks (the half-life of the active form of vitamin D (1,25(OH)2D) is only 4–6 h [4]. For the commodity of this paper, we will use the term “vitamin D” instead of “25(OH)D” when talking about the general concentration of this vitamin in the sample.

### 2.4. Statistical Analysis

In assessing the normal distribution of variables, the Kolmogorov–Smirnov test was used, and the mean (standard deviation—SD) and median [interquartile range—IQR] were reported accordingly. For continuous variables, the one-way ANOVA test was performed for those following a Gaussian distribution, whereas the Mann–Whitney U test was used for non-Gaussian distributed variables in order to compare groups. Categorical variables were presented as percentages and were tested with a chi-square test or Fisher’s exact test as required.

In order to identify the independent association between various parameters and the diagnosis of severe CAD, an univariable binary logistic analysis was conducted. Subsequently, significant variables identified from the univariable analysis were included in a multivariable binary logistic regression analysis using a backward conditional method to select independent predictors.

All statistical analyses were conducted utilising SPSS Statistics (IBM^®^ Corp., Armonk, NY, USA, version 24). A *p* ≤ 0.05 was considered statistically significant.

## 3. Results

### 3.1. Population

The study enrolled 1484 patients with AMI. Table 1 shows the baseline clinical and demographic characteristics of the whole population and after stratification by gender.

The mean age of the cohort was 66.3 (11.5) years and one-third of the patients (28.2%) were female. Most patients presented with a first ischemic event (83.5%), 86.2% of them had CAD, and 32.9% had severe CAD.

The median vitamin D concentration was 17.3 [10.3—24.7] ng/mL. Hypovitaminosis D was identified in 59.7% of the patients, whereas 23.6% of the cohort had severe hypovitaminosis D.

### 3.2. Gender Differences in the Cohort

Female patients were older (*p* < 0.01), presented more severe clinical symptoms upon hospital admission (Killip > 1, *p* = 0.03) and exhibited a worse GRACE score at six months (*p* < 0.01) compared to their male counterparts. Concurrently, male patients exhibited a higher prevalence of cardiovascular risk factors and a more frequent history of previous ischemic events (*p* < 0.01).

Regarding CAD diagnosis, males exhibited a higher prevalence of CAD (89.6% vs. 77.6%, *p* < 0.01) and severe CAD (34.9% vs. 27.7%, *p* < 0.01) compared to females.

**Figure 1:**
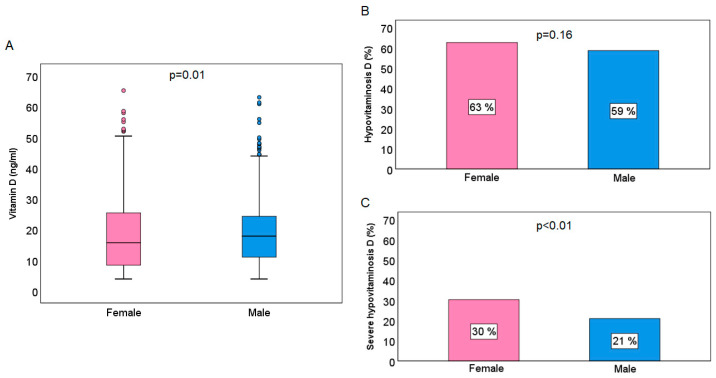
Graphical presentation of the mean values of vitamin D (**A**), hypovitaminosis D (**B**) and severe hypovitaminosis D (**C**) prevalence according to gender.

As illustrated in Figure 1, the female gender had significantly lower vitamin D levels compared to the male gender (15.7 [8.4–25.4] ng/mL vs. 17.9 [11–24.3] ng/mL, *p* = 0.01). With regard to hypovitaminosis D, the prevalence was found to be higher in females than in males, although this difference was not statistically significant (females vs. males, 63% vs. 59%, *p* = 0.16). On the other hand, a significantly higher prevalence of severe hypovitaminosis D was observed in females compared to males (30% vs. 21%, *p* < 0.01).

### 3.3. Vitamin D and CAD Severity in Female Gender

Female patients with hypovitaminosis D compared to those without exhibited a more severe Killip class, a higher prevalence of comorbidities, such as DM and anaemia, and had a higher GRACE score at six months (Table S1).

**Figure 2:**
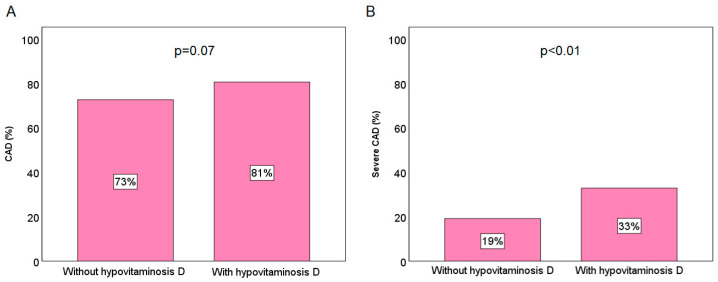
Prevalence of CAD (**A**) and severe CAD (**B**) according to the hypovitaminosis D presence in the female population.

Regarding the CAD diagnosis, a tendency towards a higher prevalence of CAD was identified among female patients with hypovitaminosis D (81% vs. 73%, *p* = 0.07), while severe CAD was found to be significantly more prevalent in female patients with hypovitaminosis D compared to those without (33% vs. 19%, *p* < 0.01), as shown in Figure 2.

Logistic regression multivariable analysis reveals that hypovitaminosis D significantly increased the risk of severe CAD (OR: 1.85 [1.14–3.01], *p* = 0.013), together with DM and CKD, adjusted for age, cholesterol and body mass index (Table 2). In addition, women with both hypovitaminosis D and DM were observed to have a risk of severe CAD development that was more than three times higher than that of women who lacked both (OR: 3.59 [1.84–6.99], *p* < 0.01).

### 3.4. Vitamin D and CAD Severity in Male Gender

**Figure 3:**
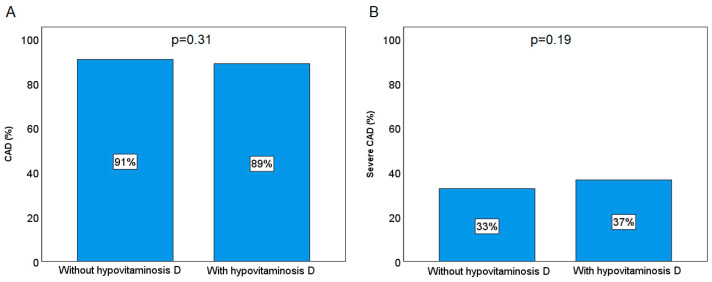
Prevalence of CAD (**A**) and severe CAD (**B**) according to the hypovitaminosis D presence in the male population.

As with women, male patients with hypovitaminosis D exhibited a more unfavourable clinical presentation at the time of admission and were more likely to present with cardiovascular risk factors and comorbidities, as detailed in Table S1. No significant difference was observed in the prevalence of CAD and severe CAD between male patients with and without hypovitaminosis D (*p* = 0.31 and *p* = 0.19, respectively; Figure 3). Further analysis does not corroborate the hypothesis that vitamin D status plays a role in the development of severe CAD, as demonstrated in the female sub-cohort.

## 4. Discussion

This study has several strengths. To the best of our knowledge, this is the first study that includes a real-world cohort of consecutive patients with AMI, comprising both STEMI and NSTEMI, that has demonstrated gender differences in the association between hypovitaminosis D and CAD severity. We found that, among women, hypovitaminosis D significantly increased the risk of severe CAD. Furthermore, the coexistence of hypovitaminosis D and DM increased the risk of severe CAD threefold. We would like to point out that our study provides valuable and novel insights that complement the limited existing data on this gender disparity topic. Indeed, despite the existence of numerous studies indicating lower vitamin D levels in female subjects compared to their male counterparts [7,8,9], which was also confirmed in our study, few have investigated gender differences in the impact of vitamin D levels on CAD prevalence and severity [2,3]. One of the rare studies on this issue is research by Verdoia et al. that involved 1858 patients who underwent elective coronary angiography, comprising patients with stable angina/silent ischemia, acute coronary syndrome and dilated cardiomyopathy/arrhythmias/valvular disease, in which was observed that women had significantly lower levels of vitamin D and that severe hypovitaminosis D was independently associated with the risk of CAD prevalence and severity of the disease in women. In men, however, this association was only observed with regard to the risk of CAD prevalence and not with regard to CAD severity [2].

In the last two decades, numerous studies have pointed out that a considerable proportion of individuals with cardiovascular disease (CVD) exhibit suboptimal levels of vitamin D. In fact, several studies have already demonstrated the association between lower vitamin D levels and CAD diagnosis and poor outcome [3,5,15,16]. Moreover, there is a clear association between reduced levels of this steroid hormone and an elevated risk of developing CVD, even among individuals without a previous history of the disease [17]. For instance, in the Health Professionals Follow-up Study, male participants with no CVD history but with hypovitaminosis D exhibited a twofold increased risk of ischemic events during 10 years of follow-up compared to male participants with optimal levels of vitamin D [18]. Moreover, the Framingham Offspring Study yielded similar results, indicating that individuals with no history of CVD but with severe hypovitaminosis D are at a markedly elevated risk of developing CVD [15].

Furthermore, in addition to the correlation between vitamin D and the incidence of CAD [19], there is also a noted association between vitamin D levels and the severity of CAD. In this context, it is worth mentioning an interesting study conducted by Norousi et al., which enrolled 732 consecutive patients with stable, unstable angina and MI. The study found a correlation between low vitamin D levels and an elevated risk of CAD and the extent of coronary artery disease [3]. It is also noteworthy that when the participants in this study were divided according to their respective diagnoses, there was a statistically significant difference in the mean vitamin D levels. More precisely, despite the majority of enrolled participants having hypovitaminosis D, it is notable that patients with myocardial infarction exhibited lower vitamin D levels than those with stable or unstable angina [3].

The rationale for the adverse effects associated with hypovitaminosis D can be attributed to the multiple beneficial roles of this steroid hormone. Indeed, vitamin D has emerged as a cardioprotective marker due to its involvement in the regulation of inflammation, oxidative stress, endothelial function, glucose homeostasis, vascular tone and stiffness, and blood pressure [4]. Specifically, vitamin D exerts multiple roles through both genomic and non-genomic actions [4]. Upon the binding of vitamin D to the vitamin D receptor (VDR), VDR moves to the nucleus, regulating the expression of genes involved in proliferation, cell cycle progression, differentiation, DNA repair and apoptosis [4]. Vitamin D has been demonstrated to suppress the nuclear factor kappa enhancer of light chain (NF-κB) activity through the physical interaction of the VDR with IκB kinase β, which is a component of the transduction mechanism for NF-κB activation [20]. This results in the alteration of numerous processes in which NF-κB is involved, including reduced production of pro-inflammatory cytokines, such as tumour necrosis factor-α (TNF-α), interleukin-1β (IL-1β) and interleukin-6 (IL-6) [20].

Therefore, the lack of vitamin D results in the promotion of a pro-inflammatory state, which leads to a reduction in nitric oxide (NO) concentration due to the TNF-α-mediated downregulation of endothelial NO synthase (eNOS) activity [4,21]. Consequently, lower NO synthesis impairs vessel integrity and vascular contraction, which are extremely important for proper endothelial function [22]. Furthermore, low vitamin D levels are also associated with a reduction in NO synthesis via other distinct mechanisms. For instance, the regulation of eNOS occurs at various stages of protein production, including gene transcription, post-transcriptional and post-translational levels and at the level of protein–protein interactions in response to diverse stimuli [21]. Calcium waves and oscillations and calmodulin regulate eNOS activity and NO production [23]. Given that vitamin D regulates the intracellular homeostasis of calcium, hypovitaminosis D also contributes to the impairment of NO synthesis in this way [4]. Furthermore, it is important to add here that both VDR and 1α-hydroxylase, a crucial enzyme in vitamin D activation, are present not only in endothelial cells but also in vascular smooth muscle cells (VSMCs) and cardiomyocytes regulating, among various processes, calcium homeostasis and contraction [24]. Therefore, all the processes involved in endothelial dysfunction are highly intertwined and interconnected at multiple levels.

In addition, regarding calcium homeostasis, in instances of low calcium levels in the bloodstream, resulting from low intestinal absorption due to hypovitaminosis D, the parathyroid hormone (PTH) enhances renal calcium reabsorption and prompts the release of calcium from the bones, thereby compromising bone strength [25,26]. On the other hand, the PTH and vitamin D are closely correlated since the PTH stimulates the metabolism for activation of vitamin D in the kidneys, whilst vitamin D exerts a negative feedback mechanism on PTH secretion.

**Figure 4:**
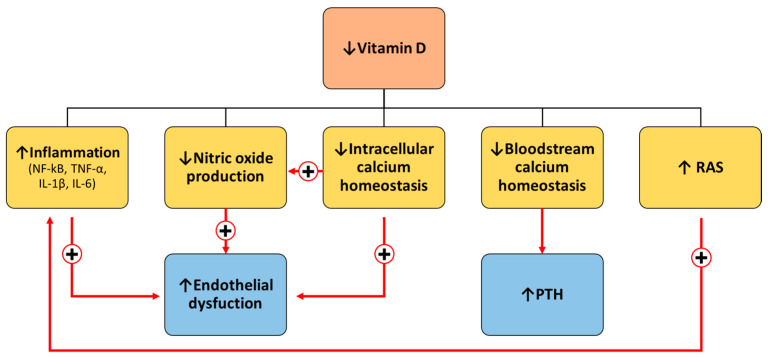
Physiopathological mechanism associated with hypovitaminosis D. IL-1β, interleukin-1β; IL-6 interleukin-6; NF-κB, nuclear factor kappa enhancer of light chain; PTH, parathyroid hormone; RAS, renin–angiotensin system; TNF-α, tumour necrosis factor-α.

Therefore, considering all these processes, we can say with a certain amount of confidence that vitamin D protects against atherosclerosis [3,5,15,16]. Furthermore, a deficiency in vitamin D has been demonstrated to result in the overexpression of renin, thereby activating the renin–angiotensin system (RAS). This, in turn, has been linked to the development of renal and cardiovascular injuries [27]. In addition to cardiovascular diseases, research has shown that hypovitaminosis D is associated with many other chronic diseases, including metabolic conditions, such as obesity or diabetes [4,28] (Figure 4).

Finally, regarding the gender differences in vitamin D levels [7,8,9], there are several reasons that can explain why women are more prone to hypovitaminosis D than men, with hormonal differences between genders representing one such reason [28]. Oestrogen, for example, may enhance the production of vitamin D-binding protein, affecting circulating levels of vitamin D, while testosterone induces vitamin D catabolism. Consequently, hormonal changes during menopause can impact vitamin D synthesis and metabolism [28]. The aforementioned reasons may account for the diminished concentration of vitamin D observed among postmenopausal women in our study, which was concluded on the basis of the mean age of the participants. Furthermore, the differences in body composition between men and women can affect vitamin D levels. Women tend to have higher levels of body fat compared to men, especially during and after menopause when the accumulation of visceral fat is accelerated. As vitamin D is a fat-soluble hormone, it can be sequestered in adipose tissue, potentially leading to lower circulating levels of vitamin D in women, as it may be less readily available for use [28]. In addition to gender differences, other features, such as genetic factors, ageing, skin colour, diet and modern (sedentary) lifestyles, are the elements that may influence vitamin D levels [28,29]. As a consequence of ageing, skin becomes less efficient at synthesising vitamin D upon sun exposure, and many postmenopausal women may have low vitamin D levels due to a reduced dietary intake, impaired gut absorption and/or limited outdoor activity [28,29]. However, it is essential for postmenopausal women to ensure they have adequate levels of both oestrogen [30] and vitamin D [31] to support their bone health and overall well-being. This often involves a combination of strategies, including hormone replacement therapy to address oestrogen deficiency under medical supervision, along with dietary changes, supplementation and safe sun exposure to maintain optimal vitamin D levels [28,30,31].

Regarding the gender difference in the association between vitamin D values and CAD severity, it can be concluded that hypovitaminosis D contributed to the development and severity of the disease, synergistically with menopause, which is already a cardiovascular risk factor [30] in our sub-cohort of women with AMI. Specifically, oestrogen plays a cardioprotective role by exhibiting vasodilator and hypotensive effects through the modulation of NO concentration in the endothelium [30]. Therefore, oestrogen and vitamin D share molecular pathways modulating NO synthesis. In addition, in a study that included the general Swiss population, an inverse association was observed between vitamin D levels and cardiovascular mortality in women and between vitamin D levels and cancer mortality in men [32]. Furthermore, our study indicates that menopausal women who have hypovitaminosis D and DM, which affects glucose homeostasis and further exacerbates oxidative stress, thus leading to an inflammatory state [4], are at a higher risk of severe CAD.

Finally, caution is needed when extrapolating findings, such as an association between vitamin D and CAD development and severity. In particular, observational studies cannot establish causality, and observed associations may be influenced by confounding factors [33], such as the presence of dyslipidaemia, DM or a high body mass index (BMI), coexisting CKD and, finally, reverse causation [4,5]. Moreover, randomised controlled trials (RCTs) investigating the effects of vitamin D supplementation on CAD outcomes have produced mixed results, which further deepen the incomprehensibility [29,34]. More precisely, while several studies have reported null effects of vitamin D supplementation on cardiovascular outcomes [35,36,37], others have shown a significant association of vitamin D supplementation with reduced high-risk plaque burden and reduced mortality risk [38,39]. The heterogeneity of study results may be due to differences in the study populations, baseline vitamin D status, study designs in terms of vitamin D doses, duration of supplementation and the outcome measures assessed [4]. For instance, in some trials, some of the participants had optimal baseline vitamin D levels, so supplementation had no effect. In other cases, participants had a diagnosis of severe CAD or AMI; therefore, supplementation had no effect due to the patients’ already serious overall condition and severe heart damage. Considering vitamin D supplementation as a preventative measure, rather than after an AMI, as in DM settings, may be worth exploring. Furthermore, studies on vitamin D supplementation often fail to consider important patient characteristics, such as age or BMI, which can significantly influence vitamin D intake and metabolism, in addition to baseline hormone levels [4]. Finally, when conducting RCTs, it is crucial to consider the optimal dosage of vitamin D for each participant and monitor their hormone levels serially. Studies have demonstrated a J- and U-shaped association between vitamin D and poor prognosis, indicating that both excessively high and low levels of vitamin D are equally dangerous [40,41,42,43]. Therefore, further research is needed to understand these inconsistencies better in order to introduce vitamin D supplementation as a preventive and/or treatment strategy for CAD in the general population.

### Limitations

This study has a few limitations. This is a single-centre study. While this may limit the generalisability of the findings, it does offer the advantage of ensuring homogenous measurements and consistent treatment during hospitalisation. Lastly, information about dietary habits or indoor/outdoor activity was not available.

## 5. Conclusions

In conclusion, in this study that involves a real-world cohort of patients with AMI, STEMI and NSTEMI, hypovitaminosis D significantly increased the risk of severe CAD only in women and not in men. Furthermore, the coexistence of hypovitaminosis D and DM increases the risk of severe CAD threefold in women only. Despite the fact that numerous studies have shown that women are more likely to have hypovitaminosis D than men and that hypovitaminosis D is associated with CAD, there has been little research to investigate the possible gender differences in this association.

This study offers valuable and novel insights that enhance the limited existing data on this gender disparity topic. Nevertheless, more studies are needed to further explore the molecular mechanisms by which vitamin D may influence CAD development and severity differently in men and women due to the influence of sex hormones. Lastly, studies aimed at proving the beneficial effects of vitamin D supplementation in terms of reducing mortality risk and disease development and progression yield conflicting results. Therefore, further well-conducted research, including RCTs, with an adequate representation of women, is necessary to clarify these inconsistencies in order to help determine the potential benefits of vitamin D supplementation in preventing and managing CAD, with an emphasis on an individualised approach.

## Figures and Tables

**Table 1:** Clinical and demographic characteristics of the whole population at admission and stratified by gender.

	Overall (n = 1484)	Female (n = 419)	Male (n = 1065)	*p*-Value
Age (years)	66.3 (11.5)	70.1 (11.4)	64.7 (11.2)	<0.01
BMI (kg/m^2^)	26.2 [24–29]	25.5 [22.2–29.2]	26.5 [24.3–29.4]	<0.01
SBP/DBP on admission (mmHg)	135.3 (24.1)/80 [70–85]	135 (24.2)/80 [70–80]	135.4 (24.1)/80 [70–90]	0.77/<0.01
Heart rate on admission (bpm)	75 [65–85]	75 [66–85]	74 [64–84.5]	0.03
Atrial fibrillation (%)	7.2	7	7.3	0.85
Left bundle branch block (%)	4.2	4.4	4.1	0.77
Diagnosis (%)				0.23
NSTEMI	36.1	38.4	35.1	
STEMI	63.9	61.6	64.9	
Killip > 1 (%)	18.6	22.2	17.2	0.03
Hypertension (%)	65.5	67.3	64.4	0.33
Diabetes mellitus (%)	24.5	20.3	25.8	0.03
Smoking (%)	45.5	31.5	51	<0.01
Dyslipidemia (%)	55.1	57.3	54.3	0.30
Positive family history (%)	24.9	26.6	24.3	0.39
Chronic kidney disease (%)	8.7	6.2	9.7	0.03
Peripheral artery disease/Carotid vasculopathy (%)	7.1/8.2	6.4/9.1	7.4/7.8	0.51/0.53
History of AMI/PCI/CABG (%)	17.5	12.6	19.3	<0.01
History of stroke/TIA (%)	5.6	6.4	5.3	0.38
Anaemia (%)	29.4	31	28.8	0.39
Uric acid (mg/dL)	5.8 [4.8–6.9]	5.1 [4.2–6.4]	6 [5.1–7.1]	<0.01
Fibrinogen (mg/dL)	328 [275–399]	342 [283–400]	322 [271.8–398.3]	0.04
Total cholesterol (mg/dL)	184 [154–218]	199 [158–230]	181 [151–213]	<0.01
HDL cholesterol (mg/dL)	43 [36–51]	49 [41–58]	41 [35–49]	<0.01
LDL cholesterol (mg/dL)	119.4 (40.5)	124.51 (43.6)	117.5 (39.1)	0.01
Triglycerides (mg/dL)	111 [83–150]	110 [83.75–143]	112 [82–153]	0.29
hs-TnI (ng/L)	15,685 [3595–56,580]	9132 [1927.5–44,642.5]	19,671 [4836.8–62,141]	<0.01
HbA1c (%)	5.9 [5.6–6.5]	5.9 [5.6–6.4]	5.9 [5.6–6.6]	0.67
GFR (mL/min/1.73 m^2^) on admission	81.4 [64.1–99.4]	76.4 [58–94.3]	83.9 [66.4–101.1]	<0.01
GFR < 60 mL/min/1.73 m^2^ on admission (%)	20.4	26	18.1	<0.01
GRACE score at 6 months	132 [110–154]	139 [114.3–161]	130 [109–153]	<0.01
Left ventricular ejection fraction (%)	51.6 (10.9)	52.27 (11.4)	51.4 (10.6)	0.19
Mitral insufficiency (%)	57.8	65.1	55.1	<0.01
Therapy at admission (%)				<0.01
Medical therapy	19.1	29.8	14.8	<0.01
PCI	70.7	62.8	73.8	<0.01
CABG	9.6	7.2	10.6	0.04
First PCI and then CABG	0.6	0.2	0.8	0.46
Right coronary artery (RCA) > 50% (%)	54.9	50.1	56.7	0.02
Left anterior descending artery (LAD) > 50% (%)	65.5	57.8	68.5	<0.01
Left main coronary artery > 50% (%)	23.2	20.5	24.3	0.11
Circumflex artery (LCx) > 50% (%)	35.8	28.9	61.5	<0.01
Ellis C (%)	17.1	15.3	17.8	0.27
Bifurcation (%)	0.7	0.5	0.8	0.46
CAD (%)	86.2	77.6	89.6	<0.01
Severe CAD (%)	32.9	27.7	34.9	<0.01
No. of vessels > 50% (%)				
0	13.8	22.4	10.4	<0.01
1	30.9	29.6	31.4	0.51
2	27.4	25.1	28.4	0.2
3	17.9	14.1	19.4	0.02
4	10	8.8	10.4	0.36
Vitamin D (ng/mL)	17.3 [10.3–24.7]	15.7 [8.4–25.4]	17.9 [11–24.3]	0.01
Hypovitaminosis D (%)	59.7	62.5	58.6	0.16
Severe hypovitaminosis D (%)	23.6	30.3	20.9	<0.01
NYHA class on discharge (%)				0.62
I	85.9	84.7	86.4	0.40
II	12.2	12.9	11.8	0.56
III	2	2.4	1.8	0.46

Legend: AMI: acute myocardial infarction; BMI: body mass index; CABG: coronary artery bypass graft; CAD: coronary artery disease; DBP: diastolic blood pressure; GFR: glomerular filtration rate; HbA1c: glycosylated haemoglobin; HDL: high-density lipoprotein; hs-TnI: high-sensitivity Troponin I; LDL: low-density lipoprotein; NYHA: New York Heart Association; PCI: percutaneous coronary intervention; SBP: systolic blood pressure.

**Table 2:** Predictors of severe CAD in female patients with AMI.

Predictors of Severe CAD	OR (95% CI)	*p*-Value
Hypovitaminosis D (Yes vs. No)	1.85 (1.14–3.01)	0.013
Diabetes mellitus (Yes vs. No)	1.88 (1.13–3.17)	0.016
CKD (Yes vs. No)	1.99 (1.24–3.22)	0.005

Variables: hypovitaminosis D, diabetes mellitus, CKD, age, cholesterol and body mass index.

## Data Availability

The original contributions presented in the study are included in the article/Supplementary Materials; further inquiries can be directed to the corresponding author.

## Supplementary Materials

The following supporting information can be downloaded at: https://www.mdpi.com/article/10.3390/jcm13226792/s1, Table S1: Clinical and demographic characteristics of the female and male population at admission stratified by the presence of hypovitaminosis D.

## Abbreviations

25(OH)D	25-Hydroxyvitamin D
AMI	Acute Myocardial Infarction
BMI	Body Mass Index
CAD	Coronary Artery Disease
CKD	Chronic Kidney Disease
DM	Diabetes Mellitus
IL-1β	Interleukin-1β
IL-6	Interleukin-6
NO	Nitric Oxide
eNOS	Endothelial NO Synthase
NSTEMI	Non-ST-Segment Elevation Myocardial Infarction
NF-κB	Nuclear Factor Kappa Enhancer Of Light Chain
RAS	Renin–Angiotensin System
RCTs	Randomised Controlled Trials
PTH	Parathyroid Hormone
STEMI	ST-Segment Elevation Myocardial Infarction
TNF-α	Tumour Necrosis Factor-α
VDR	Vitamin D Receptor
VSMCs	Vascular Smooth Muscle Cells
