# Supplementary material for: The Co-Existence of Hypovitaminosis D and Diabetes Mellitus Triples the Incidence of Severe Coronary Artery Disease in Women
Source: J Clin Med. 2024 Nov 12;13(22):6792. doi: 10.3390/jcm13226792 (PMC11594877; doi:10.3390/jcm13226792)
Supplement: Supplementary file 1 [file jcm-13-06792-s001.zip › jcm-3278406-supplementary.pdf]

Table S1. Clinical and demographic characteristics of the female and male population at admission stratified by the presence of hypovitaminosis D.

	Female patients			Male patients		
	**Non-Hypovitaminosis D (n=157)**	**Hypovitaminosis D (n=262)**	**p-value**	**Non-Hypovitaminosis D (n=441)**	**Hypovitaminosis D (n=624)**	**p-value**
Age (years)	69.1 (11.2)	72 (11.5)	0.13	64.4 (10.5)	65 (11.6)	0.34
BMI (kg/m^2)	24.9 (4.3)	26.4 (4.8)	**0.01**	26.8 (3.7)	27.5 (4.7)	0. 11
SBP/DBP on admission (mmHg)	136.7 (22.7)/ 80 [70 - 80]	133.9 (24.9)/ 75 [70 - 80]	0.25/0.19	133.3 (23.6)/ 80 [70 - 90]	136.9 (24.3)/ 80 [70 - 90]	0.01/0.23
Heart rate on admission (bpm)	74 [65 - 84]	75 [66 - 86]	0.29	72 [62 - 80]	75 [65 - 85]	**0.03**
Atrial fibrillation (%)	8	6.2	0.66	6.5	7.9	0.5
Left bundle branch block (%)	4.4	4.5	1	2.8	5.0	0.16
Diagnosis (%)			0.61			0.47
NSTEMI	40.1	37.4		33.8	36.1	
STEMI	59.9	62.6		66.2	63.9	
Killip >1 (%)	17.2	25.2	**0.05**	13.8	19.6	**0.02**
Hypertension (%)	64.3	69.1	0.33	61.5	66.8	0.08
Diabetes mellitus (%)	13.4	24.4	**0.01**	20.6	29.5	**<0.01**
Smoking (%)	28	33.6	0.28	47.4	53.8	0.05
Dyslipidemia (%)	51	61.1	0.05	54.9	53.8	0.76
Positive family history (%)	22.9	28.6	0.21	24.9	23.9	0.72
Chronic kidney disease (%)	5.7	6.5	0.84	8.4	10.6	0.25
Peripheral artery disease/Carotid vasculopathy (%)	3.8/6.3	8/11.5	0.1/0.15	4.5/6.8	9.5/8.6	**<0.01**
History of AMI/PCI/CABG (%)	8.3	15.3	**0.05**	20.4	18.6	0.48
History of stroke/TIA (%)	3.8	8	0.1	4.8	5.6	0.58
Anaemia (%)	19.1	38.2	**<0.01**	24	32.1	**<0.01**
Uric Acid (mg/dL)	5 [4.1 - 6.1]	5.2 [4.2 – 6.6]	0.13	6 [5 – 6.9]	6 [5.1 – 7.2]	0.18
Fibrinogen (mg/dL)	336 [271.5 - 399.5]	345.5 [290.5 - 401]	0.13	308 [259 - 367.5]	333 [279.3 – 414.8]	**<0.01**
Total cholesterol (mg/dL)	195 [161 - 230.5]	203 [158 - 230]	0.66	181 [154.5 - 211]	181 [148 - 216]	0.81
HDL cholesterol (mg/dL)	52 [43 - 59]	47.5 [39 - 58]	**0.01**	42 [36 - 50]	40 [34 - 47]	**<0.01**

LDL cholesterol (mg/dL)	123.5 (41.9)	125.36 (45.1)	0.71	116.7 (36.4)	118.1 (41)	0.83
Triglycerides (mg/dL)	100 [77 - 129]	116 [89 – 150.5]	**<0.01**	110 [79 - 154]	114 [84.5 – 153]	0.14
hs-TnI (ng/L)	7730 [1645 - 30321]	10405 [2430 - 49029]	0.17	19988.5 [5188.8 - 59050]	19277 [4712.5 – 67750.5]	0.81
HbA1c (%)	5.9 [5.6 - 6.2]	6 [5.6 - 6.4]	0.12	5.8 [5.5 - 6.4]	6 [5.6 - 6.7]	**<0.01**
GFR on admission (mL/min/1.73m^2)	76.1 [61.7 - 94.8]	76.4 [57 - 94.3]	0.61	83.6 [67.2 - 100.1]	83.9 [65.1 - 102.3]	1
GFR<60 mL/min/1.73m^2 on admission (%)	22.3	28.2	0.21	16.4	19.4	0.23
GRACE score at 6 months	132 [110 - 153]	143 [1219.5 - 164]	**0.04**	131 [111.5 - 153]	129 [108 – 151.3]	0.54
Left ventricular ejection fraction %	54.5 (9.7)	53 (12.3)	0.22	53 (9.9)	52 (11.1)	0.79
Mitral insufficiency (%)	65.7	64.8	0.91	57.1	53.5	0.27
Therapy at admission (%)						
Medical therapy	31.4	27.8	**0.01**	12.5	16.5	0.08
PCI	55.5	60.4	**0.02**	78	70.8	**<0.01**
CABG	11.7	11.3	0.69	8.6	12	0.09
First PCI and then CABG	1.5	0.4	0.38	0.9	0.6	0.72
Right coronary artery (RCA) >50% (%)	45.9	52.7	0.08	52.8	59.5	**0.02**
Left anterior descending artery (LAD) >50% (%)	52.2	61.1	**0.05**	71.2	66.7	0.12
Left main coronary artery >50% (%)	15.3	23.7	**0.02**	22	26	0.15
Circumflex artery (LCx) >50% (%)	22.9	32.4	**<0.01**	39.7	37.7	0.52
Ellis C (%)	17.1	14.2	0.44	18.8	17	0.46
Bifurcation (%)	0	0.4	0.33	0.7	0.9	1
N. of vessels >50% (%)						
0	27.4	19.5	0.6	9.3	11.2	0.31
1	32.5	27.9	0.32	32.7	30.4	0.45
2	22.9	26.3	0.44	29.5	28.6	0.5
3	10.8	16	0.14	20.2	18.9	0.61
4	6.4	10.3	0.17	8.4	11.9	0.07
NYHA class on discharge (%)						

I	89.7	81.7	**0.03**	88.4	85	0.12
II	9	15.3	**0.07**	10.7	12.6	0.39
III	1.3	3.1	0.33	0.9	2.4	1

Legend: AMI: acute myocardial infarction; BMI: body Mass Index; CABG: coronary artery bypass graft; CAD: coronary artery disease; DBP: diastolic blood pressure; GFR: glomerular filtration rate; HbA1c: glycosylated hemoglobin; HDL: high-density lipoprotein; hs-TnI: high sensitivity Troponin I; LDL: low-density lipoprotein; NYHA: New York Heart Association; PCI: percutaneous coronary intervention; SBP: systolic blood pressure.
